# Penetrating Posterior Urethral Injuries: Case Report and Management Strategies

**DOI:** 10.1155/2024/7839379

**Published:** 2024-07-26

**Authors:** Seyed Sajjad Tabei, Brandon Lippold, Wesley Baas, Gregory Murphy

**Affiliations:** ^1^ Division of Urology Department of Surgery University of Cincinnati College of Medicine, 231 Albert Sabin Way, Cincinnati, Ohio 45267, USA; ^2^ Division of Urologic Surgery Washington University School of Medicine in St. Louis, 4960 Children's Place, Campus Box 842, St. Louis, Missouri 63110, USA

**Keywords:** gunshot wound, penetrating wounds, posterior urethra, urethral diseases, urotrauma

## Abstract

Penetrating posterior urethral trauma from gunshot wounds (GSW) is rare and requires prompt treatment to minimize complications. Data regarding the management of such cases is scarce in the literature and poorly addressed in the guidelines. Different management approaches exist, including urinary diversion with immediate versus delayed urethroplasty/fistula repair. We present our case series to add to our experience to the literature. Three patients aged 18–44 presented with ballistic posterior urethra injuries from GSW. Initial management involved urethral catheter placement, with one patient requiring operative placement of urethral and suprapubic catheters (SPTs). Complications included recurrent membranous stricture, urinary retention, rectourethral fistula, and erectile dysfunction (ED). Posterior urethral injuries from GSW are complex as they can be either isolated or affect adjacent organs. Bladder, ureteral, and urethral injuries must be ruled out. Unlike bladder neck injuries, immediate urethroplasty/fistula repair would be very challenging and not advised for standard prostatic or membranous injuries. Urethral catheter or suprapubic tube is recommended and can result in fistula closure and urethral patency. It is critical to maintain close follow-up with the patient due to the possibility of stricture recurrence. Urethroplasty in a delayed fashion can be very successful.

## 1. Introduction

Penetrating injuries to the posterior urethra are rare yet require prompt diagnosis and treatment to minimize long-term complications. Gunshot wounds (GSW) and explosions from improvised explosive devices during wartime are known to have caused this type of injury. Classic symptoms of urethral injury may include voiding disability, blood at the meatus, and a full bladder [1].

In addition to different characteristics of tissue injury in blunt injuries and those resulting from ballistic projectiles, conflicting ideas exist regarding the optimal management in such scenarios. The possibility of multisite injury arising from penetrating projectiles further complicates management options [2]. Often rectal injuries and rectourethral fistula can occur. The American Urological Association (AUA) and European Association of Urology (EAU) Guidelines are unclear in addressing how these penetrating situations should be managed specifically [3, 4]. While primary endoscopic realignment is controversial in Pelvic Fracture Urethral Injuries (PFUI), it is unknown how penetrating injuries respond to similar management. Owing to the healing process following urethral injury, complications such as urethral stenosis, strictures, fistulae, and erectile dysfunction (ED) may arise after the initial healing phase [5].

Herein, we present three cases of penetrating trauma to the prostatic and membranous urethra and suggest management strategies for these injuries.

## 2. Methods

We queried the electronic medical record (EMR) at our institution to identify patients over the age of 18 who presented between 2018 and 2021 with penetrating trauma to the bladder neck/trigone, prostate, or posterior urethra. Penetrating trauma was defined as any foreign object piercing the skin that caused damage to deep structures. PFUI were not included. Each patient's chart was reviewed for presentation, workup, immediate and delayed management, complications, and outcomes. All patients who met this inclusion criteria were added to this study. All procedures performed in this study were in accordance with the ethical standards of the institutional and/or national research committee(s) and with the Helsinki Declaration (as revised in 2013). Written informed consent was obtained from the patients for the publication of this case report and accompanying images.

## 3. Case Descriptions

### 3.1. Case 1

A 24-year-old male presented to an outside emergency department with a single GSW entering the left buttock and exiting near the right pubic ramus. He complained of blood at the urethral meatus and urine leaking from his GSW. A CT scan showed tissue damage near the bulbar and membranous urethra and a retrograde urethrogram (RUG) demonstrated no contrast traversing the membranous urethra. The patient was taken for cystoscopy by the referring urologist who demonstrated complete transection of the distal prostatic urethra. An 18 Fr Foley catheter was able to be passed through the transection over a wire. He additionally had a suprapubic catheter (SPT) inserted. Both catheters were removed 4 weeks postoperatively. Four months later, he developed a dense 6 Fr membranous urethral stricture and his referring urologist performed a direct vision internal urethrotomy (DVIU). Four months later, the patient had recurrence of this stricture (1 cm at membranous urethra) and was referred to our center and underwent a successful posterior urethroplasty (excision and primary anastomosis). He is voiding without complaint or recurrence at 3 years.

### 3.2. Case 2

**Figure 1:**
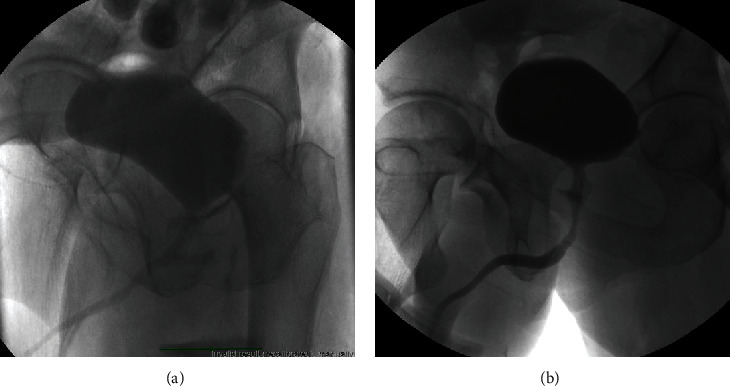
Case 2: (a) VCUG with rectourethral fistula. (b) VCUG after 4 weeks of catheter drainage and fecal diversion.

An 18-year-old male presented to our emergency department with a single GSW entering the superior anterior right thigh and exiting at the left perianal area. He complained of urinary retention and the urge to defecate and had profuse rectal bleeding. One attempt at urethral catheterization was unsuccessful by emergency room staff. CT angiogram was done at the time of hospitalization to rule out vascular injuries and showed rectal injury, space of Retzius hematoma, and free air at the base of the penis. He initially underwent right internal iliac artery embolization for rectal bleeding. He subsequently underwent a diverting loop colostomy and urethral catheterization. An intraoperative RUG and cystogram confirmed a rectourethral fistula in the prostatic urethra (Figure 1). An 18 Fr council-tip catheter was placed over a guidewire and remained in place for 4 weeks with subsequent resolution of symptoms. At his most recent follow-up visit, the patient reported an inability to obtain an erection since his original injury, and 100 mg of sildenafil was prescribed. There has been no evidence of stricture recurrence at 2 years of follow-up.

### 3.3. Case 3

**Figure 2:**
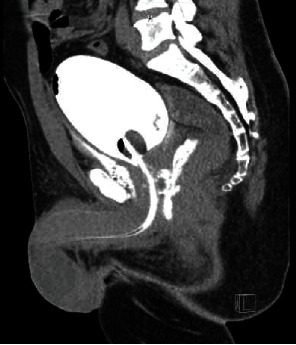
Case 3: sagittal CT cystogram showing extraperitoneal contrast extending from the prostatic urethra.

A 44-year-old male presented to an outside emergency department with a GSW entering the right inguinal region and exiting the left buttock. A 16 Fr Foley catheter was successfully placed with the return of dark red urine. Blood was also noted on the digital rectal exam. CT cystogram showed extraperitoneal contrast extending from the prostatic urethra and traumatic injury to the rectum and right inguinal canal. He was taken to the OR for diverting colostomy and inspection of the right testicle, which appeared viable. His Foley catheter remained in place for 6 weeks, and a subsequent VCUG showed no injury. He has no evidence of recurrence at 6 months follow-up (Figure 2).

## 4. Discussion

Firearm-related morbidities have become a significant healthcare concern in the United States [6]. On a daily basis, an average of 329 individuals in the United States seeks medical attention in emergency departments due to GSW [7]. The experience of Operation Iraqi Freedom (Iraq War) and Operation Enduring Freedom (War in Afghanistan) also reveals that 3%–4.6% of veterans suffered from genitourinary trauma while serving in combat [8]. Attempts have been made to report the prevalence of GSW based on the anatomical components of the genitourinary tract [9]. However, there appears to be a scarcity of multi-institutional data on penetrating posterior urethral trauma caused by gunshots and other types of ballistic projectile impact. The current AUA guidelines are heavily concentrated on the management of urethral trauma in the setting of a PFUI. The recommendation is to repair uncomplicated penetrating urethral injuries but offers little definition of “uncomplicated.” The EAU guidelines do not address penetrating urethral injuries, especially GSW, as a distinct entity.

Anatomical knowledge is essential in cases of gunshot trauma to the lower urinary tract. Studies have shown that 75% of bullet-induced urethral injuries occur in the setting of upper thigh projectile entry, regardless of whether the injury is anterior or posterior [2]. Interestingly, reports from the American Civil War indicate soldiers who were lying down or kneeling when holding a rifle were prone to urethral injuries after being shot in the perineal or buttock regions [10]. The anatomy of the posterior urethra could be disrupted in the setting of penetrating traumatic injuries such as GSW and shrapnel from explosions [11]. Presentations of penetrating posterior urethral injury vary but may include blood at the urethral meatus, inability to void due to reflex retention, and a palpable bladder [12]. However, the absence of blood at the meatus does not exclude urethral injury, as two of our cases lacked this symptom [13]. Other less common symptoms include perineal and scrotal ecchymosis [14]. However, it is reasonable that physicians evaluate the urinary symptoms along with neighboring anatomical sites in gunshot victims with extra care due to multiorgan involvement injuries. Victims of penetrating trauma such as GSWs or stab wounds are more likely to have associated injuries requiring immediate intervention. In fact, gastrointestinal (GI) injuries occur in 80%–100% of patients with penetrating GU injury [2, 9, 15]. Despite reports of the digital rectal exam's low sensitivity in the diagnosis of blunt urethral trauma (2%), this exam is required in penetrative cases to rule out perforation of structures adjacent to the posterior urethra and rule out a “high-riding” prostate [2, 16]. In our experience, two patients had a rectal injury damage requiring ureteral reimplantation. Only one of three cases complained of blood at the urethral meatus, and one complained of urine leaking from his GSW. Atypical presentations require vigilance in patients with pelvic GSWs or otherwise high suspicion for penetrating GU injury.

The AUA's guideline on the management of urethral traumas indicates that retrograde urethrography should be done as the initial step following the presence of blood at the meatus. Workup in the nonemergent setting may include RUG, or cystogram in those with suspicion for bladder injury or CT urogram to rule out ureteral injuries. We recommend ruling out bladder and ureteral injuries when penetrating injuries to the posterior urethra are suspected as the bladder and ureters are in close proximity to the prostate. Missed bladder and, in particular, ureteral injuries can lead to significant morbidity if not diagnosed in a timely fashion. RUG was particularly helpful for diagnosis in cases 1 and 2. Computed tomography is useful in emergent or more complex situations, as in our other cases. A study on 212 patients found that conventional cystography and CT cystography were similar in sensitivity and specificity in detecting bladder ruptures. That being said, CT cystography has the benefit of providing adjacent anatomical detail including the ability to track the path of the bullet. CT scans performed on gunshot patients may not necessarily yield conclusive results for evaluating the genitourinary tract due to the possible absence or displacement of the projectile remnant from the area of interest [3, 17]. However, our experience shows that in the setting of civilian gunshot injuries, we are able to obtain a clear picture of the path of the bullet and the adjacent injured organs with a CT scan. This may be due to different mechanical and velocity characteristics between military (high velocity) and civilian projectiles (low velocity). These cross-sectional imaging modalities can simultaneously evaluate for associated injury which is extremely helpful. Treatment was largely guided by imaging in our patients, and we concur with guideline statements that a RUG should be performed in all patients with suspected urethral injury [3].

Data on treatment for patients with PFUI is relatively robust. In contrast, treatment for patients with penetrating urethral trauma is somewhat variable depending on symptoms, the extent of damage, and associated injuries. In the trauma setting, vascular injury and hemodynamics obviously take priority over urethral intervention. While urinary drainage is not absolutely required in the initial hours of a trauma, excessive urinary retention or extravasation can lead to complications so must be established once the patient is stabilized [18].

We recommend the use of a 16 Fr Foley catheter for initial placement via the urethra. If this is unsuccessful, endoscopic catheter placement is an appropriate next step. Placement of a SPT is warranted if a urethral catheter cannot be established but rarely necessary if a urethral catheter is placed. We encourage urethral catheterization when feasible, as it may reduce the risk of fistula and possibly stricture formation [18, 19].

Similar to PFUI, we recommend against immediate (within 48 h) urethroplasty for penetrating posterior urethral injuries, as do European guideline statements, due to the risk for severe bleeding from pelvic hematomas and technical difficulty secondary to acute inflammation [18, 20]. Our experience confirms urinary drainage alone as a viable initial management strategy. Two patients with prostatic injuries healed their fistula and did not develop urethral stricture, avoiding the need for future surgery. These cases were classified as partial injuries, whereas the stricture requiring DVIU and ultimately posterior urethroplasty occurred in Case 1 (complete urethral injury). Pericatheter RUG or VCUG can be done to determine if the injury has sufficiently healed which in our cases took 4–6 weeks. Close follow-up is recommended to rule out stricture and fistula formation.

Complications in the penetrating trauma population mainly include urethral strictures and ED with incontinence and fistulas being less common. Tausch et al. found that 2 of 15 patients who underwent urethroplasty for penetrating posterior urethral injury had stricture recurrence [11]. Reports of ED rates in PFUI vary widely, while Tausch et al. found 6 of 19 patients with penetrating posterior urethral injuries suffered from ED. As in our Case 2, ED is a result of the original injury and not subsequent treatment. Fistulas are also commonly reported in the literature with variable success in management [2, 15, 21–23]. While these are often managed surgically, our experience in Cases 2 and 3 suggests that conservative management of rectourethral fistulas may be reasonable in select cases. These fistulas have the advantage of occurring in healthy tissue rather than an irradiated field as is usually seen with rectourethral fistulas, and as such, it makes sense that conservative management would be more successful in this cohort.

There is no agreed-upon ideal time to perform subsequent fistula or stricture surgery should they develop. In patients who are critically ill, prolonged management with SPT drainage may be necessary before delayed urethroplasty. Otherwise, we recommend urethroplasty after 3 months to ensure there has been some healing of tissue planes [24]. If the injury is at the membranous urethra, standard posterior urethroplasty can be done. If the injury is intraprostatic or bladder neck, robotic approaches would be preferred with either bladder flap or buccal graft reconstruction needed. Total prostatectomy for penetrating posterior urethra trauma has historically been used in wartime and some civilian settings, but this practice is rarely necessary except in extreme cases [11]. Should a rectourethral fistula develop, there are many approaches, and the reconstructive surgeon should perform whatever they are most comfortable with. We have found transperineal fistula repair with gracilis flap interposition to be the best option in our hands.

## 5. Conclusion

**Figure 3:**
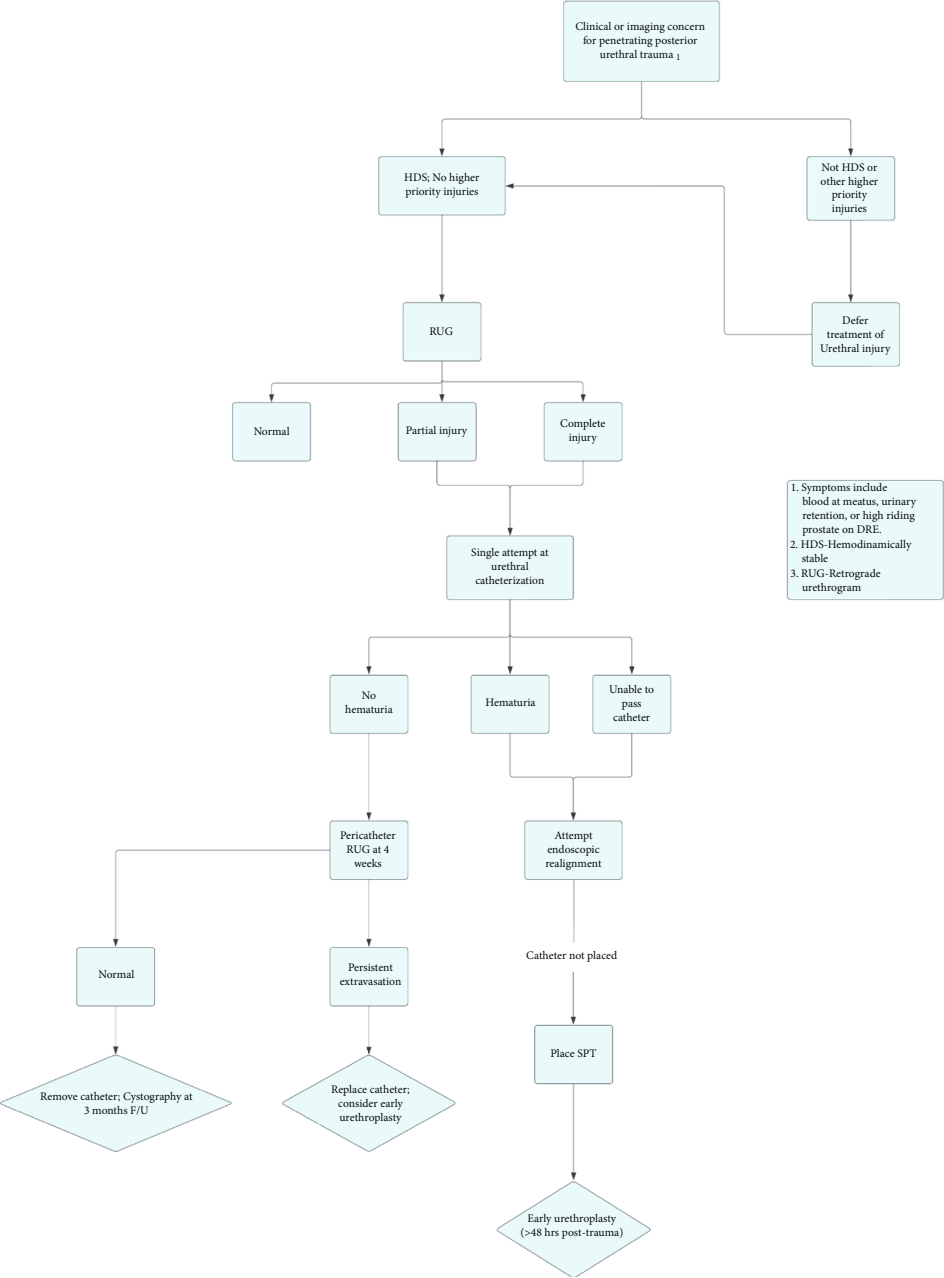
Proposed algorithm for treatment of penetrating posterior urethral trauma.

Literature regarding the penetrating posterior urethra is extremely limited, as these injuries are rare. The present case series provides more context for the management of these patients and their outcomes. Overall, our patients were managed more conservatively than those in the literature, with only one ultimately undergoing urethroplasty. Given the limited literature and our experience, we created an algorithm for the management of these injuries (Figure 3). Considering the challenging nature of posterior urethral reconstruction, a Foley catheter or suprapubic diversion should be offered initially with delayed urethroplasty reserved in cases that do not show adequate healing. However, penetrating urethral trauma remains rare, and management varied, necessitating further study, especially from centers with high volume of such injuries.

## Data Availability

Data is available upon request.
